# On Retrogressive Motions in Birds Produced by the Application of Cold to the Cervical Spine, with Remarks on the Use of That Agent as an Aid in Physiological Investigations

**Published:** 1867-03

**Authors:** S. Weir Mitchell


### ON RETROGRESSIVE MOTIONS IN BIRDS PRODUCED BY THE APPLICATION OF COLD TO THE CERVICAL SPINE, WITH REMARKS ON THE USE OF THAT AGENT AS AN AID IN PHYSIOLOGICAL INVESTIGATIONS.

By S. WEIR MITCHELL, M.D.

At long intervals during several years, I have made use of various degrees of cold as a means of research in experiments upon the nervous system, and, especially, its central ganglia.

Until recently I have been unable to control it, so as to secure from its employment all the advantages which at one time I was led to look for.

Of late, however, the interesting and valuable method of causing local anæsthesia by cold, invented by Dr. B. W. Richardson, of London, has enabled me so to improve upon the plans which I formerly followed, so as to lead, in one direction, at least, to results of the utmost interest, and I believe of entire novelty.

Were my time completely at my own disposal, I should have preferred to wait until I had developed and explained these results more perfectly to my satisfaction. Unfortunately I should have been obliged in this case to await the return of another summer, and I have therefore thought best to lay before my fellow-workers, in this branch of science, the conclusions already attained; trusting to them and to my own future labors to complete what I have in part effected.

Various methods have been made use of to study functions by injuring or destroying the organs to whose integrity they have been supposed to owe their existence. For this purpose mutilation or ablation has been employed. In other cases poisons has been given to abolish for a time the action of those portions of the centres or nerves upon which certain drugs have been thought to exert an influence. This latter mode is even more uncertain than the one about to be proposed, and has besides obvious inconveniences upon which it is hardly necessary to dwell.

During the spring and summer of 1863, Dr. Morehouse and I engaged in a prolonged research on the cerebro-spinal fluid, and the influence of pressure in producing convulsions. In the course of these experiments the following observations were made. As I was led by them to engage in the experiments which are the subject of the present paper, I think it best to copy them at length from my note books. I am the more willing to do so, since they possess an interest in themselves, and are, I believe, rather novel in character.

*Experiment 1.—June 6th.* Large rabbit cut down in middle line, and made a minute opening through the membrane which fills up the occipito-atloid space.

A tube twelve inches long, and two millimetres wide, open at both ends, was drawn to a point at one extremity, and expanded funnel like at the other. The lips of the small end were rounded carefully and made to flare outwards a little. This extremity was then slipped through the hole in the membrane which it accurately closed, when drawn gently outwards, so as to bring the flange of the tube against the inside lip of the opening. The tube was then held upright, and water temperature 66° F. was poured into its upper end. It was possible in this manner to pour into the tube, and through it, into the spinal canal, at least *half an ounce* of fluid. At a certain point, usually when there was a pressure of ten inches in the tube, convulsions ensued and checked the experiment.

The injectment thus made, displaced the blood in the spinal vessels, and caused bleeding from any exposed veins of the cord or head. Died next day. Medulla oblongata softened.

*Expt. 2.* In two rabbits we repeated this observation, injecting water at 100° F., when the largest amount was borne before general convulsions took place.

Water at 120° F. Less pressure was borne and convulsions came sooner. When water at 32° F. was employed we found that the spasms followed almost instantly upon the introduction of the first few drops of fluid.

The convulsions which ensued were very remarkable. The animal rolled, turned, leaped, shivered, and in fact exhibited every variety of convulsive action in remarkable perfection. This effect seemed to be due to the direct influence of cold on the medulla oblongata, since like effects were produced when a morsel of ice was laid gently upon that organ, or when it was exposed and iced water was dropped upon it.

This very striking experiment led me at a subsequent time to test the possibility of suppressing the functions of central ganglia by the use of cold.

It is scarcely necessary to do more than allude to the attempts which I have made to effect this end. At intervals during the summers of 1863 and 1864, I made many applications of ice and ice and salt in bags, to the spines and brains of rabbits, Guinea-pigs, and kittens. Nearly all of these experiments failed to give striking results. In some cases, as where I tried to freeze the part without removing the overlying tissue, no marked phenomea ensued. In others, where I placed the freezing mixture on the bony spine itself, or on the head, feebleness alone was produced. In no instance did I make use of birds until the spring of 1866, from which time my present experiments date.

At the period last referred to, it occurred to me to try whether Dr. Richardson's application of the atomizer to local anæsthesia might not enable me to effect the purpose in which I had previously failed. I also reflected that birds might answer better than quadrupeds, on account of the thinness of their skulls, and the comparative isolatson of some of the central organs—such as the cerebellum, and the optic tubercles. On the other hand, birds offered an embarrassment in the high temperature which their bodies present, and which, of course, makes it difficult to freeze any portion very deeply. The heat of the pigeon, for example, is frequently nine degrees above that of man.

Notwithstanding this drawback, the change in the animal used, and the employment of Richardson's method enabled me for the first time to get satisfactory results. Among the most striking were those which I obtained while attempting to freeze the necks and heads of pigeons, and it is these which I propose here to relate at length.

The method in this research was much the same in every case, and will exact very little description; Richardson's atomizer was necessary when ether was used. In general, however, rhigolene was employed with the aid of a cross jet of air—such as is made use of in atomizing water. This is mentioned particularly because the form of apparatus which is best for ether does not answer so well with rhigolene.

In some instances the atomized fluid was thrown upon the skin, in others on the bony case of the nerve-centres, and more rarely on the nerve substance previously laid bare, or merely guarded by a thin patch of caoutchouc. The animals employed were pigeons, chickens, rabbits, cats, and frogs.

*Expt. 3.—June 29th, 1866.* I threw a jet of rhigolene upon the back of a pigeon's head. The time not stated in my notes. He shivered towards close of freezing. When released he walked away, shook himself, cleaned his feathers and bill, etc.

In one minute he began to move backwards suddenly, as if by his own will. Before each motion of this kind he squatted close to the floor, and as he began to move, threw his tail up and his head down, and to one side. The series of motions had the appearance of a spasm. In the intervals he sought a corner, and sank into a stupid condition, or else walked about as usual until either a backward motion overtook him or he fell anew into stupor. From this he was easily aroused, and was then to appearance in full possession of all his faculties. The backward movements at length became visible only as sudden checks which overtook the pigeon now and then while walking forwards.

This experiment offered certain novel and curious facts. The delay before any noteworthy phenomena were seen, the sudden onset of a strange form of convulsive movement, the state of partial stupor apt to follow, and finally the healthy appearance of the animal in the interval, reminded me of the symptoms of certain epileptic cases, and made me hasten to repeat the observation.

*Expt 4.* Same pigeon frozen two minutes a little lower down, so as to more exactly influence the cerebellum. Released he walked away, well and strong, pluming himself, and picking his breast, a common motion. Within twenty seconds the first backward motion took place; at the same time an increasing difficulty of movement overtook him. He walked as if drunk, lifting his legs very high, in a curious fashion. Five minutes from the freezing he still had frequent backward motions, which gradually lessened. At the tenth minute he began to exhibit the attacks of stupor.

*Expt. 5.* Used rhigolene over the cerebellum of a young pigeon for about four minutes. Released, he could not stand, but rocked to and fro, and threw himself from side to side with general movement of all the muscles, but no orderly execution of any series of movements. Second minute, ran forward naturally for the first time; tried to fly through the window, and uttered the cry common to young pigeons. At the third minute he began to turn somersaults backwards, and did so for half a minute, when he stopped a moment, and again continued to turn over backwards. Then came a period of stupor, broken at the fifth minute by continuous backward turns. He still cried at intervals, and the somersaults gave place to backward movements which were very rare, when at the ninth minute I ceased to observe the bird. Three days later the pigeon was perfectly well, except that he had a small slough over the most prominent point at the back of the skull.

Up to this time I had seen nothing very novel, excepting the temporary creation of spasms, such as in some shape were known to occur when the cerebellum was mechanically injured. The following experiment opened a newer field:—

*Expt. 6.* A pigeon whose cerebellum had been previously frozen many times was chilled on both sides of *the neck* during one minute by a double jet of rhigolene, at the level of the tenth cervical vertebra, just above the junction of the neck of the body. When released, he became at once the sport of violent general convulsions. They were very complete, and offered no other striking character for a few moments; then there were occasional backward somersaults, or an effort in this direction. At the second minute the backward walking motion began to be seen, as the general spasms and the somersaults ceased. The stupors also appeared, and alternated with the runnings backward until within an hour the animal gradually recovered its usual health.

If the results above noted had followed the chilling of the cerebellum only, I should have been little surprised; but nothing in our previous knowledge of physiology led me to look for general convulsions or backward spasms from injury to the spine below the medulla oblongata. After witnessing this remarkable result I repeated the experiment again and again upon a number of pigeons, with precisely the same results, sometimes obtaining backward somersaults, and at others only retrograde movements, alternating with periods of stupor and intervals of apparently full control of all the normal movements.

At another part of this essay I shall describe more minutely the character of these abnormal motions. At present, I pass on to consider the limits of the region which, being chilled, will give rise to them. I began at the cerebrum and made the following experiments, which I briefly state:—

*Expt. 7.* Pigeon previously frozen several times, but now perfectly well. Rhigolene spray on skull over cerebrum for four minutes, guarding the cerebellum with a card, and also preventing, in a similar way, all inhalations of the vapor, a precaution which was taken whenever the freezing was near to the head. Released at 6.44½. His head rolled about a little. His general movements were uncertain, and he walked away, seeking a corner. One and a-half minutes. Continuous backward somersaults, with short intervals of rest and stupor. Sixth minute. Same phenomena. Observation ceased.

*Expt. 8.* Rhigolene on cerebral region three minutes. Pigeon in a state of profound sleep or stupor. Third minute after freezing head falls, eyes closed, weak, falls sideways, but soon tries to walk, and seeks a corner. Fourth minute, stupor, roused by falling over. Occasional spells of like nature, but less marked, and further apart. No backward movements at any time.

*Expt. 9.* Exposed cerebral lobes of pigeon; no bleeding; drew skin together over the brain, and threw rhigolene jet on it for one minute, freezing it as hard as ice. Set free, he flew straight up into the air one or two feet, again and again for three minutes; then had spells of vehement forward movement, during which he stumbled, with his neck bent forward, eyes shut, not directing his steps, and running against any obstacle put in his way. In four minutes he became more conscious, tried to fly out of window, and suddenly fell into stupor, from which a push aroused him. Eighth minute. Backward walking spasms for eight or ten steps at a time, varied with stupor. Nearly well at twelfth minute.

*Expt. 10.* After an hour froze the exposed cerebrum of same pigeon one and three-quarter minutes. Asleep; awakened somewhat in ten seconds, and had slight general convulsions. First minute, intense forward flexion of neck. Attempting to move forward he falls on his side; alternate sudden forward rushing motions and backward walkings, the latter predominating. Fifth minute. Backward motions only, but well marked. Recovery at seventh minute.

In these and similar experiments I sometimes succeeded in abolishing for a few moments the cerebral functions, and causing deep sleep. In one case the freezing went so deep as to influence the region of the copora striata, occasioning the violent forward movements which Magendie originated by ablating these parts.

Besides these phenomena, backward motions were sometimes observed, but they were never so violent as when the cerebellum or spine was directly attacked.

Since it is practically very difficult to limit the freezing so as to be sure when you freeze the cerebrum that you do not also chill the cerebellum. I am inclined to suppose that the backward motions which occur when we are acting on the former organ may really be due to an affection of the latter; at all events, it is well to remember that in the experiments here related, and in other like attempts to freeze the cerebrum, I have but once seen the backward somersaults, which are the most remarkable exhibitions of this form of compulsory activity. There is, therefore, some reason to suppose that the cerebrum is not the centre from which proceed these singular impulses.

It was next desirable to know what parts of the spinal column, upon being frozen or chilled, may give occasion to the phenomena in question.

I began by throwing rhigolene upon the skin over the medulla oblongata. The principal symptoms were great weakness, with confused movements of the wings and legs, feeble and laborious breathing, and, after a few minutes, backward walking, with rare attacks of stupor. As the part lay deep, it was difficult to avoid chilling the cerebellum. The following experiment may serve as a type of the larger number, which it is needless to report:—

*Expt. 11.* Rhigolene on skin over medulla oblongata for two minutes, the skin being drawn tightly over the spine, and the head bent forward. Released, very weak, tendency to fall to left side, shivering, feathers ruffled; on walking forward tends to fall on his bill. Third minute. Begins to show backward motions. Fourth minute. Repeated backward movements. Seventh minute. Increased feebleness, backward spells, gradual recovery, with rare fits of stupor.

In the next experiment I laid bare the bony spine just below the medulla oblongata, and threw on it a jet rhigolene, during three minutes. Released, lies on one side, breathing feebly, unable to move, pupils sensitive, reflex motions in both legs on pinching one of them. Second minute. Put on his feet, rocks to and fro, breathing more labored; gradual improvement with fits of deep stupor. Twelfth minute. Backward movements alternately with stupors; slow recovery.

*Expt. 12.* Rhigolene on neck, half an inch below medulla oblongata at third or fourth cervical vertebra three minutes. Backward spells at third minute, with incessant somersaults; previously ran about as if well; stupor; tried to enter his cage. Plumed himself. Fifth minute. Somersaults ceased, and are replaced by backward walking, varied with stupors. Recovered.

*Expt. 13.* The skin in this pigeon was drawn tightly over the spine, and a double jet of rhigolene thrown on the tenth vertebra for two and a-half minutes. Released, he ran about, apparently well. Twenty-fifth second. Marked backward spells, which were incessant until the fourth minute, when he had stupor, followed by confused general convulsions and backward walking. Fifth minute. Backward somersaults, etc. Recovery.

The spine was next acted on at the third cervical vertebra, with the same phenomena. In a second experiment at this same point a double jet was used. The feebleness and disorder of respiration were more marked, the stupors and movements were as before. Recovery perfect in both cases.

The next experiments were made at points between the middle neck and the first fixed vertebra, which itself lies a little back of the upper line of the scapula. In every case I obtained the usual series of backward movements, stupors, and intervals of perfect control. I find that no somersaults are noted in connection with these records, but as they occurred when the spine was chilled below this point, the exception becomes unimportant.

The upper interscapular line is marked by a prominent vertebra, just below which lies the first fixed portion of the spine. In a well-grown pigeon it is at least three and a-half inches below the lower level of the skull. Beneath this point the backward spells can no longer be obtained by any extent or depth of freezing.

The following notes relate one out of many experiments in which the jets of rhigolene were thrown exactly between the shoulders:

*Expt. 14.* Rhigolene jet four minutes on first fixed vertebra, at upper level of interscapular space. Released, he ran about, and in fifteen or twenty seconds sank down, shivered, and ran backwards repeatedly; during the quiet intervals he attacked another pigeon. He was next seized with stupor, then another series of backward spells, and again stupor, after which he once more assaulted the other pigeon. Recovered.

In other experiments I guarded the spine with my fingers above and below the frozen part, but still obtained, in nearly every case, the retrograted motions and the stupors.

*Expt. 15.* Froze skin with rhigolene jet one-half inch below prominent interscapular vertebra, at level of second dorsal vertebra, for four minutes. Released he walked away briskly; in half a-minute crouched, showed signs of weakness; has spells of stupor, which come on gradually, so that if he is on a perch he falls off, and is aroused by falling; well in the intervals, except that he seems weaker than common. Observed one hour, when he was well, having no longer any backward spells.

*Expt. 16.* Rhigolene jet four minutes on spine, half inch below first fixed vertebra. Ran about as usual; appeared agitated and restless, and weak in his legs; no backward spasms, no notable stupor.

*Expt. 17.* I next threw rhigolene jet on the spine, one inch above the line of the great trochanters of the femur, a point easily found. It is at the upper level of the sacrum. Froze during four minutes. Released, he ran about clumsily, tripping, intertangling his legs as he moved, and using his wings to aid progression. The movements were so futile and irregular that they presented much the same appearance as when the cerebellum has been frozen deeply or removed by the knife; no stupor, but occasional tendency to rest quiet in a corner; no backward impulsion. Recovery.

*Expt. 18.* Rhigolene jet thrown for four minutes on spine, *half* an inch above line of trochanters. Clumsy motions, great feebleness of limbs, walks crouching, now and then falling on his side. One and a-half minutes. Sank to rest in a corner, shivering; tremor of legs, especially the left leg; no backward motions or stupors. Recovered.

*Expt. 19.* Rhigolene jet on spine four minutes, at the last dorsal and first lumber vertebra, over the place at which the nerves of the legs make exit. Sudden and violent tetanic spasms of both legs and the tail, during which he fluttered about with the aid of his wings. At the third minute the spasm relaxed, and in four and a-half minutes he could walk; he remained feeble for a time, but in an hour was as well as usual.

It results from these experiments and others that at or about the fourteenth vertebra counting from above downwards, we cease to notice the backward spasms and stupors, and see only signs of weakness or of tetanic rigidity in the legs; usually these phenomena come on some time after the freezing, and reach a maximum within ten minutes.

It appears thus far that in pigeons the application of cold to the cervical spine occasions, after a brief period, peculiar backward movements, resembling those which have been previously produced by mechanical injury of the cerebellum. These abnormal actions are, in extreme cases, backward somersaults, followed by spells of backward walking, and accompanied with spasmodic movements of the head, to be fully described hereafter. In milder cases only the backward walking occurs. Both of these forms of constrained movement are met with when the cerebellum has been chilled. A further series of experiments determined that it is immaterial whether the spine be acted on behind, at the side, or in front. In fact, this organ in the pigeon is so small in diameter, and the freezing so difficult to limit, that we might naturally look for the spasms to follow an application of cold to any side of the spine.

It is indifferent whether we freeze through the skin drawn tightly over the bone, or act on the bare nerve-tissue itself.

The following is an example of the latter mode of affecting the nerve-centres:

*Expt. 20.* Exposed spinal cord of pigeon at fifth cervical vertebra, without hemorrhage of any moment, covered it with a small piece of glazed paper, and threw on this pulverized rhigolene jet for twenty seconds. Released, the bird lay twenty-five seconds breathing heavily, as if about to die, and was then suddenly seized with violent backward somersaults, which were continuous during one minute. They were followed by and finally alternated with fits of stupor, the whole ending in backward walkings. Recovery.

I was now interested to know whether the same results could be obtained by chilling the spine of other birds. For this purpose I selected the chicken, making the following very remarkable observations:

*Expt. 21.* Exposed bony spine of a large chicken-cock, and for five minutes threw jet of rhigolene upon it, at a point about three inches below occiput. Released, he walked away without any signs of stupor or sickness. In a minute he stood still, looking about him as if confused. At the second minute he began to move backwards, rotating to the left, with long, slow, and strangely deliberate steps. The rotation seemed to be due to a feebleness of the left leg. He moved in a circle of about two feet in diameter, and now and then leaped up in the air abruptly. His head was not thrown down as was always the case in pigeons, and the action had none of the appearance of spasm which it presented in that bird. After five minutes continuance of this most extraordinary motion, his steps became more awkward and irregular, as well as more rapid and spasmodic, but he now ceased to move in a circle.

At the seventh minute came the first pause, and his attacks were henceforward separated by intervals, during which he walked as usual. As the spasm came on, he slowly stooped, drooping his tail feathers, and raising his head. He seemed to have difficulty in extending his toes, which doubled under him as he stepped, and increased the awkwardness of his gait. After twenty minutes his spasms ceased.

Repetitions of this experiment gave like results, and in one case the backward movements were produced by the application of the rhigolene jet during only thirty seconds.

I have totally failed to produce these curious fits in the rabbit, the only mammal upon which I have as yet experimented.

The following analysis of observations on rabbits exhibits my results in this direction:

*Expt. 22.* Froze the bared occiput of young rabbit two and a-quarter minutes. The only symptoms were feebleness, and signs of pain, such as squealing, etc.

*Expt. 23.* Froze same point four and a-half minutes. Fell on side; kicked a good deal; apparent temporary want of power to coördinate his muscles; walked freely after a few minutes.

*Expt. 24.* Froze rabbit on spine five and a-half minutes, an inch below occiput. Released, he lay kicking violently half a minute, then rose to his feet, and fell, kicking again. Finally, he rose and fell several times without kicking, and, at length, was able to walk freely. Recovered in half an hour.

In all the other cases, four in number, I observed disordered movements, and, at the close, great weakness only.

The failure to produce in rabbits the results so readily obtained in birds appeared to indicate some previously unsuspected differences in the nervous systems of these two classes of animals, and made me desirous to see whether I should succeed any better with cold-blooded batrachians. I therefore made a number of experiments upon frogs, but without the satisfactory results I had hoped for. When the rhigolene jet is thrown upon the region of the brain, the functions of this organ are abolished for a time, and the frog exhibits the phenomena which are common to this animal when decapitated. A jet cast on the spine occasions spasmodic movements of the legs, and, at intervals, violent tetanic contractions. After a few minutes the frog is able to crawl, but does not recover the power to leap, its usual mode of locomotion, until a longer time has elapsed. Recovery is always perfect, and there are no backward movements.

When the jet is thrown on the back of the prominent eyeball, the posterior tissues of the eye are frozen without the cornea having become dim, and in this case very perfect temporary cataract is produced.

The real seat of the backward impulses in birds would seem to be at any point from the lowest cervical vertebra up to the cerebrum, whose power to originate them is at the least doubtful. Two facts, or sets of facts, led me to think it possible that it was in every case the cerebellum or the medulla oblongata, which was the organ finally responsible for the production of the spasms of retrograde motion, which we have been considering.

The first of these facts is the well-known and valuable discovery of Brown-Séquard, that in Guinea-pigs mechanical injury of certain regions of the spinal cord, from the seventh dorsal to the third lumbar vertebra, subjected the animal to fits of an epileptiform nature, which might be induced by various means, or might arise spontaneously. Moreover, these spasms began about the face, so that the spinal wound (partial section) must have produced an over-excitable condition of the nerve-centres above the point of injury.

In my pigeons, likewise, the spasms began with a flexing of the neck, etc., so that it is altogether possible that in these animals also the spinal irritation may act by causing an undue excitability of organs within the skull.

Before continuing, it may be as well to state that I do not recognize any other relation than I have here pointed out between Dr. Brown-Séquard's very brilliant discovery, and that which I am now setting forth. The fits he caused came on after some weeks had elapsed; they were seen in Guinea-pigs; they were due to mechanical injury to the dorsal or lumbar spine only, and were finally epileptiform, in all of which respects they differed from the novel facts I have observed.

The second set of facts, above alluded to, has also made me hesitate in assigning to the point of the spine injured by cold, the entire responsibility of producing the retrograde spasms.

Magendie pointed out what many other physiologists have since seen, that certain deep injuries of the cerebellum, especially in birds, gave rise to backward somersaults and movements, which were very violent, and were sometimes continuous for days, or were interrupted only for a few moments, when exhaustion was complete.

The spasms caused by cold applied to the spine were also of this retrograde nature, and I was therefore furnished with an additional motive for suspecting that they might really be due to a reflex affection of the cerebellum.

To determine the relation between backward spasms originating in mechanical injury of the cerebellum, and those due to chilling of the spine, I made a large number of experiments and observations. And first, it is to be observed, that when we chill the cerebellum, we cause convulsive movements which cannot be distinguished from those which occur when we chill the cervical spine. Either the whole of these fits then are referable to one single organ, the cerebellum, or else there is in the pigeon an extensive region, at least four inches long, and stretching from the cerebral mass to the first dorsal vertebra, any portion or section of which is capable of developing the retrograde convulsive motions when treated as I have described.

Backward somersaults and enforced backward walking from mechanical injury to the cerebellum, differ very remarkably from those produced by cold. The wound of the cerebellum must be deep to give the proper effect. The result is violent tumbling backwards, or sideways and back, with little or no respite, and certainly with no intervals of healthy activity. The spasms from cold, though often as violent, do not endure so long. They end in backward walking, and this in perfect health. I have caused them by the application of cold to the spine twenty times in one pigeon, within a few days, and still he not only survived, but ate, drank, and moved with usual vigor. Besides this, the spasms from cold alternate with attacks of stupor, in which the head falls, the eyes are shut or open, and the pigeon seems to be in a kind of lethargy. When in this state it may often be handled, or laid on its back or side, without an effort at resistance, while at any moment a loud sound or sudden motion will break the spell, and it will abruptly run backwards several feet. It was very remarkable to observe the mode in which the stupor came on. If the pigeon was on a perch or window-ledge, its head gradually fell, the body swayed backwards, and at last the feet unclasped, and the bird fell on the floor.

In the chicken the stupor was sometimes so deep that it could be held passively suspended by one foot or a wing.

The intervals of healthy activity between the times of stupor present no marked phenomena to distinguish them from the common state of the pigeon.

There are some other differences between the spasms from chilling the spine or cerebellum, and those from wounding the latter organ. In cerebellar injury the head is carried well back and high up. During the somersaults it is drawn further and further back, until the bird rolls head over tail.

In the spasms from chilled spine or cerebellum, the head is carried at ease during the intervals between the fits; but at the moment of attack the bill strikes the floor quickly, first on one and then on the other side, the head being drawn violently forwards. Even in the most terrible of the somersaults caused by cold, the head was drawn forwards, and the backward turn was produced by the action of the muscles of the legs and wings, rather than by those of the back, neck, and spine.

It remained to determine if, when the cerebellum was severely injured, or ablated, I could still obtain the typical backward spasms on freezing the spine.

I found that when the cerebellum was partially injured, so as to occasion turning, or backward somersaults, that an after application of cold to the spine, would introduce into the convulsions rare attacks of the peculiar form of retro-spasm, which were characteristic; that is to say, the pigeon would retreat, with first a spasmodic flexion of the head and neck.

When I ablated the cerebellum, so as to produce utter want of coördination, I always failed to secure the display of any backward motion, upon afterwards chilling the spine. To settle this question more thoroughly, it will be necessary to ablate the cerebellum, and keep the pigeon until it recovers the power to control its movements, as does sometimes happen.

If then it should continue to have retro-spasms upon freezing the cervical spine, we might reasonably conclude that these spasms do not exact the interference of the cerebellum—a question which, after all, I should not have thought it necessary to raise, if the peculiar retrogressive nature of the fits had not pointed to this organ, for, up to this time, as I have stated, no other has been known to possess the power to originate this special form of convulsive action.*

At present, therefore, I cannot attribute the spasms from cold to the spine alone; and, indeed, I feel strongly inclined to regard this organ as merely the point of departure of a morbid excitation which is finally translated, so to speak, by the cerebellum, into a language of its own, and thus occasions a peculiar form of compulsory movement.

While considering, in this connection, the whole train of symptoms which follows the use of cold on the spine, there is still left for consideration the state of stupor to which the animal becomes liable when so treated.

This sympton is often slight in character, but is sometimes so well marked that it resembles the deepest sleep, and even approaches the condition of coma. It then becomes plain that this is a state of system in which the cerebrum is affected, and for a time loses its functional activity. Of this there can be no possible doubt. As it was suggested, however, that the stupor might be due to the general depression of temperature caused by the freezing, I made numerous experiments to settle the question thus raised. For this purpose I chilled or froze muscular and other parts for five minutes at a time, but without obtaining stupor or spasms. Moreover, I found that both

* Elsewhere I have mentioned the medulla oblongata as having been supposed capable, when injured, of causing retro-pulsion. There is, however, some doubt on this point; but, at all events, my reasoning would apply to any organ in the head which might be presumed to have this property.

symptoms often continued unaltered, when the pigeon had regained its normal standard of temperature.

If, therefore, chilling the spine determines a marked *cerebral* disturbance, there is no reason why we might not assume, with logical propriety, that the *cerebellum* may be ultimately responsible for the backward spasms. Without being at all sure of this, I beg leave to offer the above considerations as casting some light on a point which I still regard as most obscure.

In dealing with this subject I have neglected to consider the possible share which the medulla oblongata may have in causing backward movements. Magendie considered that both this organ and the cerebellum were the seats of a normal forward impulsion. The corpora striata were, as he conceived, the centres of an opposite tendency, so that, when the two former organs were wounded, the balance was destroyed, and retrogression took place.

Apart from his theory—from which most recent biologists, excepting Vulpian, have totally dissented—it is enough to know that the medulla oblongata may give occasion to these motions, so that it is not impossible that the backward spasms which follow chilling of the spine may be finally referable either to this centre, or to the cerebellum, or to both.

I have never caused such movements by injuring the medulla oblongata, and the passage in which Magendie states the fact is a very brief one.* If, however, he be correct, and if chilling the spine can cause backward spasms by an indirect influence on higher nerve-centres, whatever I may have said in regard to the cerebellum is equally applicable to the medulla oblongata, or to any part whose direct lesion is known to produce retrograde spasms.

We have still to study the nature of the injury done to the spine or brain by cold applied through the skin or directly.

It was seen throughout my experiments, which have reached to ninety in number, that in almost every case an appreciable interval—and often a long one—existed between the close of the freezing and the access of the spasms. This period varied from a few seconds to twelve minutes. If there was any primary effect, it was merely feebleness, or disorder of movement. When I noticed these facts, I suspected at once that what is seen on the skin after freezing, repeats itself in the nerve substance. First, there is chilling and contraction of bloodvessels, and then actual freezing, which is rarely very deep. Indeed,

* Précis de Phys. Magendie, ed. 1836, p. 409.

I have found it most difficult to freeze the pigeon's entire breadth of spine. When this does occur in the upper cervical region, death by apnœa follows at once.

The freezing being over, the part thaws, and long-continued, intense congestion ensues, as any one may observe who will try the effect of the ether or rhigolene douche on his own skin.

It only remained to see if this congestion actually took place in the nerve-substance, as I suspected it must do.

To determine this point, I laid bare the cerebrum of a large pigeon, and carefully noted the color of the tissue, and the number and position of the chief vessels of the meninges. The jet of rhigolene was then used directly on the part. The visible vessels were instantly frozen, with their contents. As the part thawed, it became intensely congested, the brain darkening distinctly, and the vessels of its transparent coverings increasing in size and number, so that those which could before be seen were larger, and new ones, previously unseen, came into view.

This experiment was repeated several times, with no essentially different result.

In the last mentioned experiment I had preserved the cerebral meninges intact. On a second pigeon I repeated the observation, removing the membranes with great care. I found that several large vessels penetrating the brain from below, came to the surface, and were for the most part torn asunder in displacing the membranes. I chilled the bare cerebrum, covered with thin caoutchouc for half a minute. As it thawed, an intense congestion appeared, with numerous points of much deeper color. This condition increased during several minutes, but caused no notable disturbance of function.

In the spine of the pigeon I have thought I could see a deepened color after freezing, but of this I am not sure, owing to the small size of the cord. When, as I shall presently show, we act on this part by other irritants than cold, the resultant congestion is no longer doubtful.

The singular convulsions and stupors which I have described are therefore due, as I think, to the palsy of the vessels which have been chilled by the cold, and which may or may not have undergone previous extreme contraction. The congestion from cold, whether in the nerve tissue or the skin, is most intense at a certain time after the part has thawed, and it is then in the centres that the excitation becomes such as to determine a convulsive attack. These facts naturally incline me to regard the congestion as the essential parent of the nerve changes, which finally result in the spasm and stupor. We should not lose sight, however, of the possibility of the nerve-substance being itself directly altered by the intense cold to which it is subjected. While, therefore, I consider the congestion which does certainly occur as competent in the case, it is well to remember that the nerve-cells may be most seriously affected during the physical changes of condition, which great alternations of temperature occasion.

My success in causing spasms and other phenomena by the aid of cold, at once led me to anticipate the most interesting results from this method of research, for, if it were now in our power to create in a nerve-centre such a congestion as should allow us to study its effects, and if passing away it shouid leave the animal in a state of health such as beforehand no one could have looked for, I could not but hope that most important results in the study of pathology might thus come within our reach.

Since, however, it was very difficult to limit perfectly the influence of cold, I endeavored to see if, by other means, I could reproduce the phenomena which cold had enabled me to discover. The production of the backward spells was a very good test. If they were due to congestion, irritants, such as heat and acids, alcohol, etc., should also occasion them by causing an increased flow of blood to the spine. Besides, the local congestion thus created would be manageable, and enable me further to control the phenomena for purposes of study.

After numerous experiments I succeeded in attaining the desired end, and in causing by irritants spinal congestion and backward spasms; that is to say, the same train of symptoms which succeeded the use of cold.

*Expt. 25.* The spinal cord was exposed in a pigeon's neck, and water, at a temperature of 165° F. was thrown on it gently from a syringe; about three ounces were used. Released, it walked away, and showed no notable immediate result. After half an hour, I repeated the observation twice, using again water, temperature 165° F., and lastly, temperature 190° F. The use of the fluid in fine stream may have lowered the temperature a little, but it certainly coagulated the flowing blood and the bared muscular tissue. Yet to my great surprise the only immediate symptom was a little weakness. The pigeon was observed for an hour, and again three hours later, but exhibited no enforced motions. It was found dead the next morning.

*Expt. 26.* I dropped strong ammonia on the bare cervical cord of a pigeon. It seemed to cause pain. The bird moved about restlessly, and at last fell with enfeebled respiration, which became rapidly worse, destroying life in a few minutes after the application to the spine.

In this case the irritant was too strong and acted as a caustic. The effect of the heat (Expt. 25) I cannot explain, and will only observe that the observation was incomplete, as the pigeon was not watched constantly after the operation.

In the next observation I made use of tincture of capsicum as the irritant.

*Expt. 27.* The cervical spine was bared at the fourth cervical vertebra, and treated with six or eight drops of tincture of capsicum. I observed no results during two hours observation of the bird, except feebleness and uncertainty of gait. I had cut the posterior spinal vein in my dissection, and I was not sure how much of the symptoms might be due to loss of blood. The following afternoon, twenty-five hours later I reëxamined the pigeon. To my great satisfaction, it had marked attacks of backward motion, but no somersaults. It died forty-eight hours after the operation.

*Expt. 28.* Exposed cervical cord, at the fifth vertebra; dropped on it six drops of tincture of capsicum. As the capsicum was applied the long posterior vein of the cord, which I had exposed, but not cut, was seen to dilate suddenly, as though forcibly filled or paralyzed, and the cord visibly darkened, while a sudden hemorrhage disclosed itself on the edges of the incision. When set free, the pigeon was very unsteady, and rolled about on its feet as it walked; I then washed away the tincture with a little water and the aid of a pipette.

At the third minute, distinct backward movements were seen, with the usual dipping of the beak. At this time the spinal substance was intensely congested, but no distinct vessels could be seen. The backward movements occurred at intervals up to the seventh minute, when stupor took place. After this time the spasms took place mostly when the bird was roused from the stupor. This latter condition was not so profound as after the chilling, but lasted longer. After an hour and seventeen minutes he still had very violent backward motions, which continued for ten seconds or more at a time, when the stupor returning, interrupted them.

Twenty-four hours later this bird was well, to appearance, ate and drank as usual. The backward movements still continued, but ceased within thirty-six hours. At the end of a week the pigeon was well and active. It died five weeks later; but through an accident; no *post mortem* examination could be made.

It thus appears that the backward movements may be excited by agents, which, like tr. capsici, irritate, congest, and, perhaps, by virtue of the alcohol, chemically alter the spinal tissue. It is notable, also, that, as in spasms from cold, so in those caused by an irritant, the attacks appear only after an interval has elapsed, and are therefore not due to primary alterative influences, chemical or other.

I believe that I have made clear, in the foregoing paper, the following points: That cold may be made valuable as a means of studying the functions of nerves and nerve-centres; first, by chilling the organ down to the temperature, as yet undetermined, at which its functions cease. Secondly, by the congestion which follows, and which enables us to imitate at will a pathological condition, and thus to forward the synthetic study of neural disease.

With the aid of cold, and of the irritants which its use suggested, I have occasioned in birds, (pigeons and chickens,) a peculiar form of spasmodic or enforced movements, consisting in somersaults and sudden backward walking, such as have not been previously observed by physiologists, except when the cerebellum was mechanically injured.

I may be permitted to state, in concluding, that I regard as incomplete the research which I have described. Even the primary questions in connection with it are not all answered to my satisfaction, while so large a number of secondary points of interest have presented themselves as to make it quite unlikely that I should find time just know, among more pressing occupations, to give them the attention they will receive, I trust, from others than myself. Some, at least, of these investigations I hope myself to undertake at a future period.

In my present series of experiments I have been aided throughout by my friend, Dr. Wm. W. Keen, to whom I am much obliged for many valuable suggestions.—*Am. Journal Medical Sciences.*

